# Disruption in Wrist Literature: Identifying Paradigm-Shifting Publications

**DOI:** 10.1016/j.jhsg.2026.101090

**Published:** 2026-08-12

**Authors:** Maya D. Sinha, Hadley Grundman, Sameer R. Khawaja, Michael B. Gottschalk, Nina Suh, Eric R. Wagner

**Affiliations:** ∗ Department of Orthopaedic Surgery, Emory University School of Medicine, Atlanta, GA; † Department of Orthopaedic Surgery, Baylor College of Medicine, Houston, TX

**Keywords:** Disruption, Disruption index, Innovation, Wrist literature, Wrist surgery

## Abstract

**Purpose:**

Research in wrist surgery has advanced considerably, yet objective metrics of innovation remain limited. The disruption index (DI) offers a quantitative framework to identify papers that redirect scientific attention toward new directions, which traditional bibliometrics may overlook. Within this framework, this study aimed to examine trends in disruptive research over time. We hypothesized that DI would not correlate with citation count.

**Methods:**

A retrospective bibliometric analysis was performed using PubMed with wrist-specific search terms and limited to 14 selected orthopedic and wrist surgery journals. Each article was matched to its respective disruption scores in the publicly available Harvard Dataverse data set. Statistical analyses included descriptive statistics and Pearson correlation. Temporal, authorship, and journal-level patterns were evaluated, and the top 10 most disruptive publications were identified.

**Results:**

A total of 4,138 wrist-related publications were identified and included in the analysis. Disruption scores were narrowly distributed around zero, with a mean of –0.008 ± 0.042, and were weakly associated with total citation counts (r = 0.049, *P* < .002). The most innovative research surfaced in the 1950s (DI = 0.071) and 1960s (DI = 0.044), with scores trending toward zero over time. The journal with the highest mean DI was *Clinical Orthopaedics and Related Research* (DI = –0.0027). The most disruptive publication was “Management of Fractures of the Greater Multangular. Report of Five Cases,” (DI = 0.857), which received only eight citations. Among the top 10 most disruptive papers, the mean DI was 0.44 ± 0.16, with an average citation count of 134.7 ± 202.5 and an average of 1.8 ± 1.2 authors.

**Conclusions:**

This study applied the DI to wrist surgery literature. These findings provide new insight into how the field has progressed over time and suggest continued opportunities to pursue innovative, paradigm-shifting research.

**Type of study/level of evidence:**

Retrospective cohort study III.

Progress and innovation remain foundational to the practice of medicine, driving advancements across specialties, including wrist surgery. With rapid technological evolutions, access to emerging data and novel insights has become readily accessible. However, the exponential growth of available information has also introduced new challenges in discerning the most impactful and clinically relevant publications. The Hirschel-index (h-index), gross number of publications, and gross number of citations remain the traditional metrics used to assess research productivity, with total citation count representing the most widely used measure of publication impact.^1^, ^2^, ^3^, ^4^ Although a useful metric, citation count may not accurately reflect the true impact of scholarly works. Additionally, the number of citations may be influenced by time since publication, with older publications accruing more citations.^2^^,^^5^ Furthermore, citation counts are impacted by factors including the controversial nature of an article, having more common versus niche topics as the subject matter, and its establishment as a foundational or frequently cited reference.^6^

First introduced in economics, the notion of disruptive innovation is said to be comprised of five essential elements: associating, questioning, observing, experimenting, and networking.^7^ These core concepts may also be applied when evaluating medical literature. The impact of publications cannot be adequately assessed by solely looking at the citation count. In order for a true paradigm shift to occur, Kuhn^8^ proposes that the periods of normal science, the accepted day-to-day functioning of a field, must be completely disrupted by a period of revolution, whereby cumulative anomalies build up, prompting a breakdown and rebuilding of the field. In this vein, “disruption index” (DI) has been developed to better represent the effects of a scholarly work by quantifying the amount by which a publication presents a paradigm shift and its effectiveness in advancing a given foundation of knowledge.^2^^,^^9^^,^^10^ Within this framework, works are classified as either disruptive, changing or challenging previously held beliefs, or developmental, furthering existing information.^2^^,^^9^^,^^10^ Disruptive publications are quantified by a disruption score (DS) of >0; developmental pieces have scores <0, with the total range of scores falling between −1 and +1. Certain fields may have their own perspectives on what constitutes a disruptive article and may discuss this in literature, but DI allows for a more objective evaluation of the paradigm-shifting scholarly works. Notably, applications of this validated bibliometric tool have previously demonstrated that smaller teams are more likely to disrupt existing literature, and larger teams incrementally develop upon previous ideas.^10^

DI has only recently been explored within orthopedic literature, particularly in shoulder surgery, in addition to other medical fields.^11^ However, its application to wrist surgery remains limited, and the assessment of publication impact continues to be poorly defined. This study aimed to identify the most disruptive pieces in wrist surgery scholarly works, to evaluate the relationship between team size, DI, and citation count, and to discern the most disruptive journals in wrist surgery. We hypothesized that DI metrics would recognize uniquely disruptive wrist surgery literature when compared to works evaluated solely by citation count.

## Materials and Methods

### Study design

This study was exempt from institutional review board approval because it is a retrospective bibliometric analysis based on publicly available information and did not involve any human participants or patient data. The methods of this study are like those described by Khawaja et al.^11^

### Journal selection

Fourteen journals were selected for inclusion based on their prominence in orthopedic and wrist-specific literature. Journals included in the analysis were *Journal of Hand Surgery (European Volume), Journal of Hand Surgery (American Volume), Hand, Hand Clinics, Hand Surgery & Rehabilitation, Journal of Wrist Surgery, Journal of Orthopaedic Trauma, Journal of Bone and Joint Surgery, Clinical Orthopaedics and Related Research, Techniques in Hand & Upper Extremity Surgery, Arthroscopy, International Orthopaedics, Journal of Orthopaedic Research, Journal of the American Academy of Orthopaedic Surgeons*.

### Data collection

Wrist publications were identified using PubMed based on predefined search terms related to wrist anatomy, including “wrist", "distal radius", "carpal", "carpus", "distal ulna", "scaphoid", "lunate", "triquetrum", "pisiform", "trapezium", "capitate", "hamate", "distal radioulnar", "radiocarpal", "carpometacarpal", "TFCC", "triangular fibrocartilage complex", and "scapholunate". The search was limited to publications from 1954 to 2014 to be consistent with the timespan of the Harvard Dataverse data set. This search process identified a total of 7,452 wrist publications within this time frame, of which 4,138 (55.53%) were successfully matched to the Harvard Dataverse data set by PubMed identification number.

### DS calculation

DSs were obtained from the publicly available Harvard Dataverse data set, which includes over 42 million scientific articles from the Web of Science domain. Wu et al^10^ calculated DSs in the source data set with the formula DS = (A − B)/(C + D), where A is the number of future papers that cite only the focal article, B is the number of future papers that cite both the focal article and its references, C is the total citations received by the focal article, and D is the number of future papers that cite the focal article’s references but not the focal article. Scores range from –1 (purely developmental and never cited independently) to +1 (purely disruptive and only cited independently).^10^

### Data analysis

For each matched publication, the article title, year of publication, PubMed identification number, number of authors (team size), citation count, and DS were recorded. The top 10 most disruptive papers based on DS were recorded. To explore the characteristics of more recent research publications, a subgroup analysis was conducted of papers published from January 1, 2000, to December 31, 2014.

### Statistical analysis

Descriptive statistics, including frequencies and mean disruption indices, were calculated for the total cohort and by year, team size, and journal. Pearson’s correlation coefficient was calculated to assess the relationship between citation count and DSs. Statistical significance was set at *P* < .05.

## Results

### DS distribution

**Figure 1:**
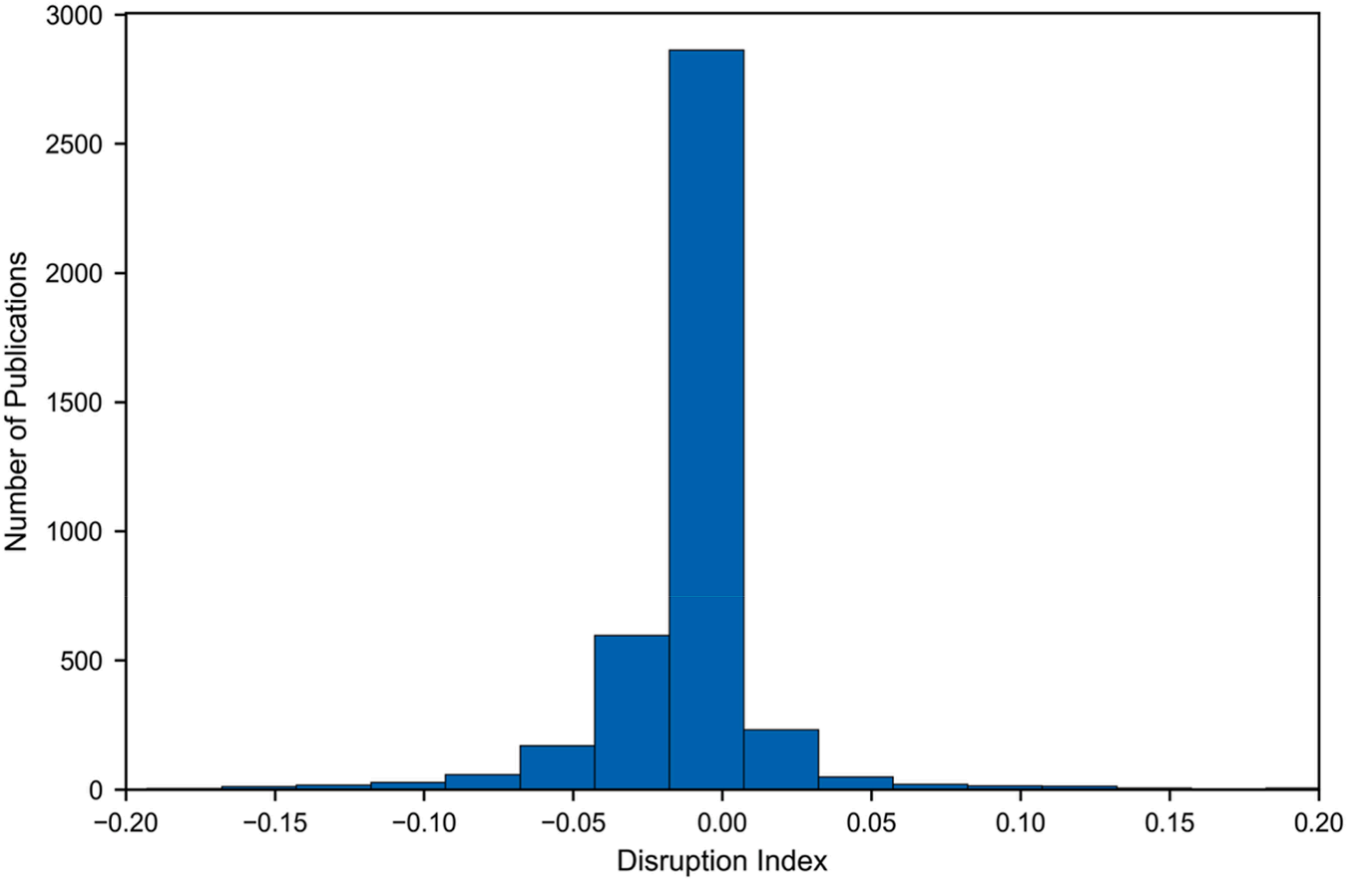
Histogram depicting the distribution of DSs.

**Figure 2:**
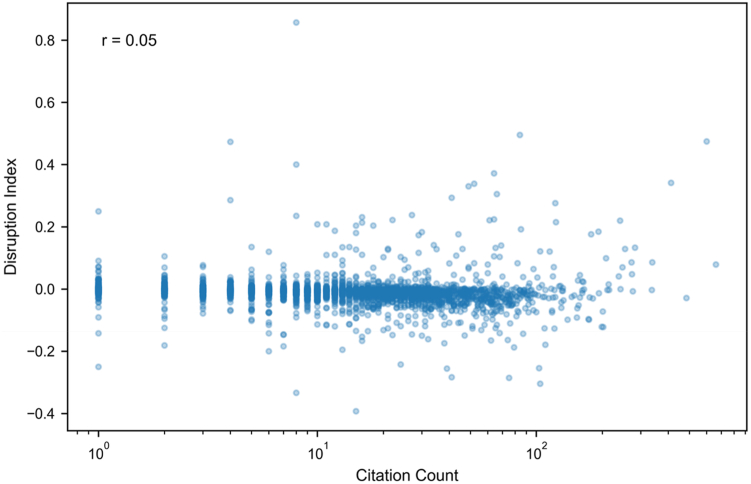
Scatterplot comparing DS and citation count.

A total of 4,138 wrist surgery articles published between 1954 and 2014 were identified and matched to their respective DSs. DSs were concentrated around zero, with 96.9% falling between –0.1 and 0.1 (Fig. 1). Across the entire data set, the mean DS was –0.008 ± 0.042, and the mean citation count was 18.0 ± 31.7. The correlation between the DI and citation count was weak (r = 0.049, *P* < .002) (Fig. 2).

### Disruption based on Journal

Across journals, mean disruption indices remained consistently near zero (Table 1). The journal with the highest volume of wrist publications, *The Journal of Hand Surgery (American Volume)*, had 1,767 matched articles with a mean DS of −0.012. *Clinical Orthopaedics and Related Research*, had the highest mean DS (–0.003) and the greatest percentage of articles with a DS greater than zero (32.9%).

**Table 1:** Disruption Index and Citation Count based on Journal

Journal	Mean Disruption Index	Mean Citation Count	N = Number of Matched Wrist Publications
*JHS (Am)*	−0.0117	14.66	1767
*JBJS*	−0.0066	43.49	606
*CORR*	−0.0027	17.64	550
*JHS (Euro)*	−0.0033	4.60	383
*Hand Clinics*	−0.0051	9.48	364
*JOR*	−0.0043	21.75	126
*Arthroscopy*	−0.0154	15.95	110
*JOT*	−0.0045	13.52	110
*Int Orthopedics*	−0.0029	10.95	83
*JAAOS*	−0.0045	13.00	43

*CORR, Clinical Orthopaedics and Related Research; JAAOS, Journal of the American Academy of Orthopaedic Surgeons; JBJS, Journal of Bone and Joint Surgery; JHS (Am), Journal of Hand Surgery (American Volume); JHS (Euro), Journal of Hand Surgery (European Volume); JOR, Journal of Orthopaedic Research; JOT, Journal of Orthopaedic Trauma*.

### Disruption trends over time

**Figure 3:**
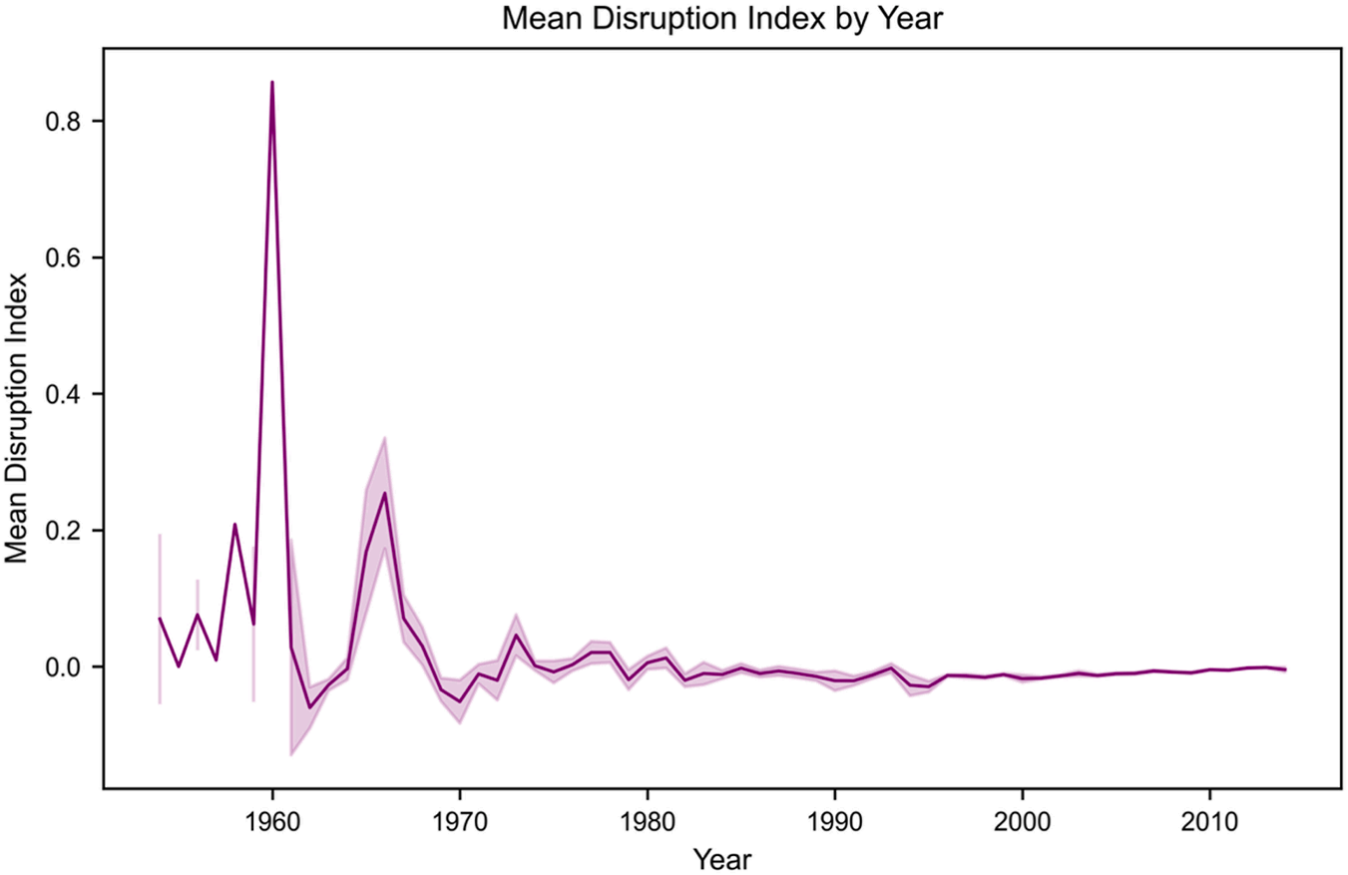
Line plot of the mean DS by year. The shaded area represents standard error.

**Figure 4:**
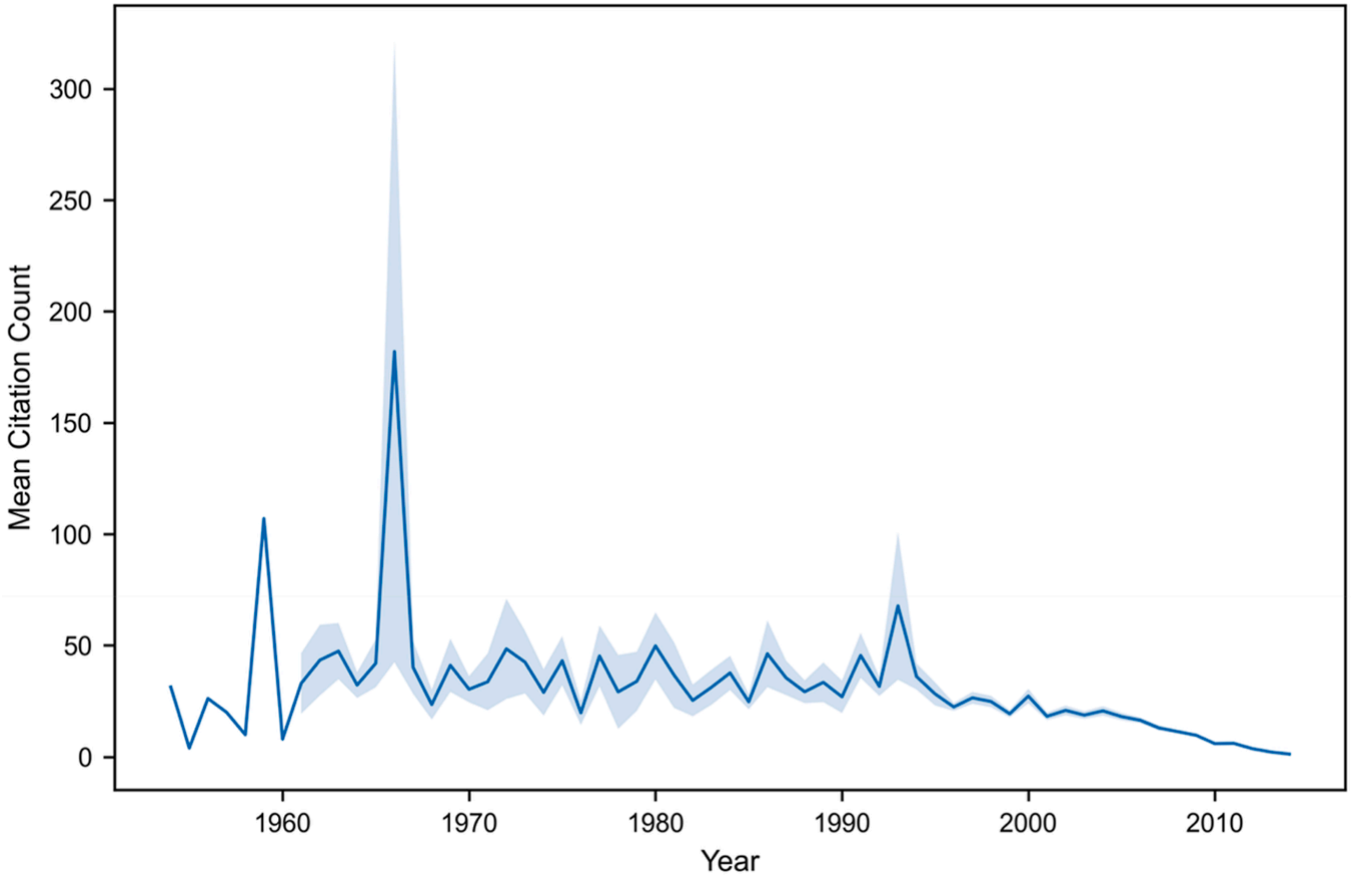
Line plot of the mean citation count by year. The shaded area represents standard error.

Mean DSs were highest in the 1950s (0.071) and 1960s (0.044), with peaks in 1960 (DS = 0.857), 1966 (DS = 0.254), and 1958 (DS = 0.208). Overall scores trended toward zero over time (Fig. 3). Analysis of citation metrics revealed that the mean number of citations per publication also followed a right-skewed distribution throughout the study period, with peak mean citations from articles published in 1966 (182.0 ± 250.7). Citation volume remained robust throughout the late 1970s and 1980s but has trended downward in the modern era (Fig. 4).

### Disruption and team size

**Figure 5:**
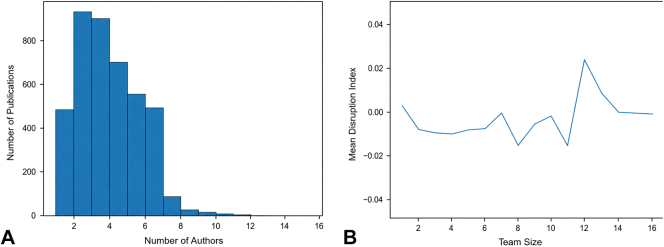
**A** Bar plot of team size distribution. **B** Line plot of mean disruption score by team size.

The mean team size for all publications was 3.5 ± 1.7 authors, with papers most often authored by three to five investigators (Fig. 5A). The mean team size for the 10 most disruptive publications was 1.8 ± 1.2 authors. Mean DSs remained close to zero regardless of team size (Fig. 5B).

### Top 10 most disruptive publications

The most disruptive publication was the “Management of Fractures of the Greater Multangular. Report of Five Cases.” (*JBJS*; DI 0.857; citation count 8). A list of the 10 most disruptive publications is provided in Table 2 and comprises articles from the *Journal of Bone and Joint Surgery* (n = 8), *Clinical Orthopaedics and Related Research* (n = 1), and *The Journal of Hand Surgery (American Volume)* (n = 1). Among this cohort, publications had a mean DI of 0.439 ± 0.16, a mean citation count of 134.7 ± 202.5, and 1.8 ± 1.2 authors. The most common decade represented was the 1960s (n = 5), followed by the 1970s (n = 2). Within the top 10 publications, the DI and citation count demonstrated a weak negative correlation (r = −0.12, *P* = .75).

**Table 2:** Top 10 Most Disruptive Wrist Surgery Publications (1954–2014)

Rank	Publication Title	Journal	Published (Y)	Citation Count	Disruptive Index
1	Management of fractures of the greater multangular. Report of five cases.	*JBJS*	1960	8	0.8571
2	Surgical treatment at the wrist in rheumatoid arthritis: a review of thirty-seven patients.	*JBJS*	1965	84	0.4957
3	The carpal tunnel syndrome. Seventeen years' experience in diagnosis and treatment of six hundred fifty-four hands.	*JBJS*	1966	600	0.4744
4	Subacute osteomyelitis in children.	*CORR*	1973	4	0.4730
5	A case of an intratendinous ganglion.	*JHS (Am)*	1985	8	0.4000
6	Indications for open reduction of lunate and perilunate dislocations of the carpals.	*JBJS*	1965	64	0.3721
7	Traumatic instability of the wrist. Diagnosis, classification, and pathomechanics.	*JBJS*	1972	412	0.3414
8	Incidence of wrong-site surgery among hand surgeons.	*JBJS*	2003	52	0.3385
9	Ulnar-nerve palsy in wrist fractures.	*JBJS*	1961	49	0.3305
10	Fractures of the carpal navicular (scaphoid): a report of 436 cases.	*JBJS*	1954	66	0.3051

CORR, Clinical Orthopaedics and Related Research; JBJS, Journal of Bone and Joint Surgery; JHS (Am), Journal of Hand Surgery (American Volume).

### Recent trends: 2000 to 2014

A subgroup analysis of the 2,790 publications released between 2000 and 2014 revealed a mean DI of 0.192 (Table 3). The correlation between disruption and citation count in this subgroup was negative (r = −0.207, *P* = .566). Within this modern cohort, the disruption indices for the top 10 most disruptive publications since 2000 ranged from 0.105 to 0.339, with “Incidence of Wrong-Site Surgery Among Hand Surgeons” (*JBJS*; DS 0.339; citation count 52) being the most disruptive.

**Table 3:** Top 10 Most Disruptive Wrist Surgery Publications (2000–2014)

Rank	Publication Title	Journal	Published (Y)	Citation Count	Disruptive Index
1	Incidence of wrong-site surgery among hand surgeons.	*JBJS*	2003	52	0.3385
2	The first description of carpal tunnel syndrome.	*JHS (Euro)*	2007	4	0.2857
3	Severely displaced scaphoid fracture treated by arthroscopic assisted reduction and osteosynthesis.	*JOT*	2000	1	0.2500
4	Subspecialty certification in hand surgery.	*CORR*	2006	8	0.2353
5	The "clenched pencil" view: a modified clenched fist scapholunate stress view.	*JHS (Am)*	2003	15	0.2037
6	The complications of Dupuytren's contracture surgery.	*JHS (Am)*	2005	34	0.1412
7	Development of the QuickDASH: comparison of three item-reduction approaches.	*JBJS*	2005	254	0.1290
8	Anatomic tilt x-rays of the distal radius: an ex vivo analysis of surgical fixation.	*JHS (Am)*	2004	21	0.1261
9	Posterior interosseous nerve terminal branches.	*CORR*	2000	11	0.1094
10	Flexor tendon fibroma as a cause of wrist triggering and carpal tunnel syndrome.	*JHS (Euro)*	2011	2	0.1053

CORR, Clinical Orthopaedics and Related Research; JBJS, Journal of Bone and Joint Surgery; JHS (Am), Journal of Hand Surgery (American Volume); JHS (Euro), Journal of Hand Surgery (European Volume); JOT, Journal of Orthopaedic Trauma.

## Discussion

The field of wrist surgery is a constantly evolving one, with thousands of publications over the last 75 years. While the number of scholarly works indicates a growing and robust subject, parsing out the most paradigm-shifting works remains challenging. Standard measures of research impact, such as citation count, altmetrics or H-index, are unable to encompass a work’s true ability to shift the paradigm. Disruption index represents a potential solution, allowing individuals to quantify whether a publication augments existing knowledge or flips it on its head. By applying this metric to wrist surgery literature, we hope to identify paradigm-shifting studies that are underappreciated for their impacts. This study aimed to identify wrist literature that was wholeheartedly disruptive.

This study identified the most disruptive wrist studies from 14 leading journals in wrist surgery literature, revealing low overall disruption in wrist surgery. Disruption showed a weak but statistically significant correlation with total citation count, highlighting the limited ability of citations to capture true innovation. Another notable trend identified was the relationship between team size and disruption. The 10 most disruptive wrist publications featured an average of only 1.8 authors, lower than the overall cohort average, which is consistent with prior work suggesting that smaller groups are more likely to pioneer innovation.^10^, ^11^, ^12^ The greatest disruption was between the 1950s and 1960s, reflecting a period of foundational discovery in wrist surgery, marked by improved understanding of wrist biomechanics and instability, microsurgical reconstruction methods, and other common wrist-related pathologies such as carpal tunnel, scaphoid fractures, and distal radius fractures.^13^, ^14^, ^15^, ^16^, ^17^, ^18^ In our modern literature subgroup, the most disruptive publications shifted greater attention toward patient outcomes and novel imaging or surgical techniques, highlighting meaningful ways that modern disruptive literature can optimize clinical practice.

The most disruptive publication was the “Management of Fractures of the Greater Multangular. Report of Five Cases.” Published in 1960, this pivotal work provided a foundational description of trapezium fractures that effectively replaced sparse preceding knowledge. Although it has a high DS, the article has been cited only eight times, highlighting how traditional citation metrics can fail to recognize research that fundamentally redirects the course of a scientific field. The second most disruptive publication was Clayton’s 1965 “Surgical Treatment at the Wrist in Rheumatoid Arthritis: A Review of Thirty-Seven Patients,” which clarified management protocols for rheumatoid wrist surgery. Although our management of inflammatory arthropathy has evolved with pharmacologic advances, this article transformed treatment at the time and provided a foundation for modern understanding of surgical management of rheumatoid arthritis at the wrist.^19^, ^20^, ^21^ These examples also support prior findings that case series studies are naturally positioned to generate highly disruptive work and introduce novel clinical insights.^22^ In the modern era, the majority of research demonstrated DSs trending toward zero. The most disruptive publication since 2000 was “Incidence of Wrong-Site Surgery Among Hand Surgeons,” ranking eighth in disruption across the overall cohort, demonstrating that innovation can thrive in an environment shifting toward consolidation.

Beyond orthopedic literature, studies in other surgical subspecialties have also examined DSs, including hand surgery, general surgery, pediatric surgery, ophthalmology, and urology literature.^10^^,^^22^, ^23^, ^24^, ^25^, ^26^, ^27^, ^28^, ^29^, ^30^, ^31^ Several of these studies have similarly shown that DSs are weakly correlated with total citation count, highlighting that citation counts may incompletely identify paradigm-shifting work.^23^^,^^24^^,^^26^^,^^28^ Hansdorfer et al^2^ reported the top 100 papers in hand surgery, with the top 30 having a DI of greater than 0.60, whereas only one wrist surgery article exceeded this threshold. This discrepancy may reflect greater consolidation within the hand surgery specialty, and future studies should examine trends in DS across other areas within orthopedic literature. However, this overall trend may not be unique to wrist surgery, as 75% of all PubMed articles from 1954 to 2014 have DSs ≤ 0.^28^ Our findings align with this global observation, suggesting that scientific research largely builds upon and refines established foundational frameworks, as described by Kuhn’s idea of “normal science,” until the field reaches a revolutionary period of innovation.^8^

As scientific literature continues to expand exponentially in the modern era, several studies have reinforced that DS captures dimensions of innovation and impact not reflected in traditional bibliometrics.^32^, ^33^, ^34^ DS serves as a complementary tool to a raw citation count by assessing the degree to which it reshapes subsequent scholarly work. Importantly, DS does not necessarily indicate clinical accuracy or acceptance.^11^ DS is influenced by broader citation behavior trends unrelated to disruption, including self-citation, preferential citation of high-impact journals, familiarity, convenience, or accessibility. Additionally, as scholarly literature continues to expand in quantity and accessibility over time, influential articles may be more likely to be highly cited alongside prior work rather than eclipsing prior work and may fail to be captured by DS. Our DS findings in wrist literature highlight a potential opportunity for renewed innovation. By identifying and analyzing disruptive studies, clinicians can better understand the field’s evolution and target opportunities for paradigm-shifting advances. Encouraging small, innovative teams, supporting novel clinical investigations, and integrating emerging technologies and novel surgical techniques may catalyze the next era of transformative innovation in wrist surgery.^35^, ^36^, ^37^, ^38^

While this study provides a comprehensive analysis of disruption within wrist surgery literature, several limitations must be considered. The analysis was restricted to 14 orthopedic and wrist journals, which may exclude potentially disruptive wrist surgery works published in other general medical or international journals. The success of this analysis was also limited by the information published on the Harvard data set, as only 55.53% of the initially identified publications were successfully matched to DSs. Many of the articles that were not matched to DSs in the database were published in more recent years or were published in specific journals, *Journal of Wrist Surgery, Hand, Hand Surgery & Rehabilitation* and *Techniques in Hand & Upper Extremity Surgery*, which may introduce selection bias. The literature search was also limited to studies from 1954 through 2014 to be consistent with the source data set, which may further exclude potentially disruptive studies. Future studies should include articles published after 2014 to evaluate more recent trends. Additionally, the distinction between wrist and hand literature can be ambiguous, and our data inadvertently included some hand and general orthopedic literature, introducing some heterogeneity. Since disruption is a temporal metric, it may take some time for a study to redirect future research and to therefore fully realize its DS potential. At the same time, older studies may tend to become outdated and therefore less frequently cited.^2^ Future studies should compare DS with other bibliometric data across other disciplines to further review their benefits in capturing various dimensions of scholarly work.

## Conflicts of Interest

Given their roles as Regional Editors for *The Journal of Hand Surgery Global Online*, Drs Suh and Wagner had no involvement in the peer-review of this article and have no access to information regarding its peer-review. Full responsibility for the editorial process for this article was delegated to John R. Fowler, MD. No benefits in any form have been received or will be received by the other authors related directly to this article.
